# Comparison of the effect between cefazolin/cefuroxime and broad-spectrum antibiotics in preventing post-operative pulmonary infections for smoking patients receiving video-assisted thoracoscopic lung surgery: a propensity score-matched retrospective cohort study

**DOI:** 10.1186/s12893-024-02329-y

**Published:** 2024-01-31

**Authors:** Guangjie Wu, Jianhua Lu, Meng Li, Dong Liu, Yan He

**Affiliations:** 1 Department of pharmacy, Tongji Hospital, Tongji Medical College, Huazhong University of Science and Technology, Wuhan, 430030 China; 2 Department of information, ZhuJiang Hospital, Southern Medical University, Guangzhou, 510280 China

**Keywords:** Cefazolin, Cefuroxime, Antibiotic prophylaxis, Video-assisted thoracoscopic surgery, Pulmonary infection

## Abstract

**Background:**

The selection of prophylactic antibiotics for preventing post-operative pulmonary infections in smoking patients undergoing video-assisted thoracoscopic lung surgery (VATLS) is not clear.

**Methods:**

In this retrospective cohort study, the outcomes of 572 smoking patients undergoing VATLS with prophylactic cefazolin/cefuroxime or other antibiotics were analyzed. Patients were classified as cefazolin/cefuroxime group and the control group. A 1:1 propensity score matching was also performed.

**Results:**

The primary outcome of the incidence of post-operative pulmonary infection did not differ significantly between the two groups (23.7% vs 30.5%, RR = 0.777, 95%CI 0.564 ~ 1.070 *p* = 0.113). Similarly, secondary outcomes including the incidence of post-operative fever, the white blood cell count and neutrophils on the 3rd day after the surgery, and time for blood routine test recovery were all found without significant difference between the two groups. In the multivariate logistic regression model, no association was found between prophylactic use of cefazolin/cefuroxime and post-operative pulmonary infections after controlling other possible confounding factors (OR = 0.685, 95%CI 0.441 ~ 1.065, *p* = 0.093).

**Conclusions:**

Prophylactic use of cefazolin/cefuroxime was not associated with more adverse clinical outcomes among smoking populations undergoing VATLS when compared with broad-spectrum antibiotics and the two drugs are still feasible for peri-operative prophylactic use for smoking population before the surgery.

## Background

Video-assisted thoracoscopic surgery (VATS) has been widely used in therapy of chest pathology. Advantages of VATS have been demonstrated in previous studies [1, 2]. Less pain, faster recovery, fewer post-operative complications, shorter length of hospital stay, and reduction of mortality than open thoracic surgical procedures have been described well. Among all the post-operative pulmonary complications, the pulmonary infection is still a major concern since it increases in-hospital mortality, intensive care unit admission and length of hospital stay in thoracic surgery [3]. Though the total ratio of post-operative pulmonary infections in patients receiving VATS is low with a range of 3–7% [2–6], the complication can lead to higher frequency of intensive care unit admission and mortality [3]. One of the measures to prevent surgical infections is antibiotic prophylaxis [7, 8]. Peri-operative antibiotic prophylaxis focuses on correct drug selection, scheme, timing, and antibiotic duration for the purpose of preventing surgical site infection (SSI) and reducing the possibility of bacterial resistance. The antibiotic chosen for prophylaxis must cover common pathogens in surgical sites. In the video-assisted thoracoscopic lung surgery (VATLS), the predominant pathogens causing post-operative pulmonary infections are *Streptococcus* and *Staphylococcus* species, followed by other gram-negative bacteria and fungal [6]. Thus, cefazolin, ampicillin-sulbactam, clindamycin as well as vancomycin are recommended for antibiotic prophylaxis during VATS [6]. In China, local guideline issued by the National Health Comission in 2015 also recommended cefuroxime as the prophylactic antibiotic for lung surgery [9].

It is widely accepted that airway bacterial colonization is associated with post-operative pulmonary infections in lung surgery with lung cancer [10–15]. In smoking population, airway colonization is different from non-smokers. In smokers, more potential pathogens were found in the nasopharynx when compared with non-smokers (0.7 vs 0.2 pathogens per patient, p<0.01) [16].

The underlying mechanism of more colonization of bacteria in smokers’ airway can be explained as smoke promotes the deposition of particles in the lower airways, where they are able to damage respiratory defense functions [11]. However, the association of these bacteria with post-operative pulmonary infections after lung surgery is still unclear. When analyzing the responsible pathogens of post-operative pulmonary infections in patients receiving lung surgery, it was found that *Haemophilus influenzae, Streptococcus pneumoniae* and *Staphylococcus aureus* were the most identified pathogens, followed by *Pseudomonas aeruginosa* and non-fermenting gram-negative bacteria [17]. Given the diversity of airway colonization in smoking patients and the pathogens responsible for post-operative pulmonary infections, it is unclear whether cefazolin and cefuroxime, which are recommended for general patients in the guidelines, are still suitable for smoking patients. For safety reasons, physicians tend to use broad-spectrum antibiotics to cover more possible colonizing pathogens for the purpose of prophylaxis in smoking patients undergoing lung surgeries which does not comply with the principle of prophylactic use of antibiotics in surgical procedures [10, 18].

Based on the backgrounds described above, we decide to conduct a retrospective cohort study to compare the effect between cefazolin/cefuroxime and other broad-spectrum antibiotics in preventing post-operative pulmonary infections for smoking patients who are to receive VATLS.

## Methods

### Participants

The data used in this retrospective cohort study were collected from the department of thoracic surgery of a tertiary hospital in Wuhan, China. Consecutive patients receiving VATLS at the department between January 1 2021 and December 31 2022 were included. The inclusion criteria: (1) patients receiving VATLS (2) ages between 18 and 70 years (3) with a smoking history (4) without a lung surgery history in the last 3 months. The exclusion criteria were as follows: (1) patients with pulmonary infections confirmed by chest imaging examinations before VATLS (2) patients transferred to an open surgery during VATLS (3) patients receiving chemotherapy before admission. Ultimately, 572 patients were enrolled in this analysis, including 152 patients receiving intravenous cefazolin (1 g, q12h, *n* = 128)/cefuroxime (1.5 g, q12h, *n* = 24) for peri-operative prophylaxis and 420 patients receiving antibiotics other than cefazolin/cefuroxime including ceftriaxone (2 g, qd, *n* = 113), ceftizoxime (2 g, q12h, *n* = 83), cefoselis (1 g, q12h, *n* = 104), levofloxacin (0.5 g, qd, *n* = 16), moxifloxacin (0.4 g, qd, *n* = 2), fosfomycin (2 g, q12h, *n* = 7), cefminox (1 g, q12h, *n* = 6), cefoperazone/sulbactam (3 g, q12h, *n* = 29), piperacillin/tazobactam (4.5 g, q8h, *n* = 60). Peri-operative antibiotic prophylaxis was started before incision, and lasted for 24 ~ 48 hours after the surgery.

### Data collection and assessment

Patients’ data were collected from the hospital information system, including demographic information, smoking history, comorbidities, nursing documents, results of laboratory tests, results of imaging examinations, medical and surgical information.

The primary outcome was the incidence of post-operative pulmonary infections during hospital stay confirmed by chest imaging examinations including X-ray or computed tomography. The imaging findings were assessed by two radiologists. The secondary outcomes included post-operative fever (defined as axillary temperature >37.3 °C measured by a mercury thermometer), white blood cell (WBC) counts and ratio of neutrophils on the 3rd day after VATLS, and the recovery time of blood routine test (blood-RT, mainly the WBC count and the ratio of neutrophils) in patients without confirmed pulmonary infections.

### Statistical analysis

All data analysis were performed using SPSS (Version 25.0, IBM Corp, New York, USA). Categorical variables were presented as frequencies with percentages, Chi-square method and Fisher’s exact test were used to test the significance. Kolmogorov-Smirnov test and Shapiro-Wilk test were used to test the normality of continuous variables according to the sample size (for sample>50 and sample ≤ 50, using K-S test and S-W test respectively). Levene-test was used to detect the homogeneity of variance. Continuous variables were then presented as mean ± SD (standard deviation) or medians with inter-quartile range (IQR) based on the normality. T-test was used for significance test of continuous variables, while Mann-Whitney U-test was used when the criteria of T-test was not satisfied. To explore the risk factors of post-operative pulmonary infections, univariate and multivariate logistic regression models were used.

To minimize the impacts of potential confounders and selection bias, the propensity score matching (PSM) method was used for baseline characteristics of the included patients. A propensity score was calculated using logistic regression, and 1:1 patient matching was performed using the nearest-neighbor matching method without replacement. Variables including respiratory comorbidities, circulatory comorbidities, pre-operative antibiotics use, respiratory function, and chronic obstructive pulmonary disease (COPD) history were matched. A caliper radius equal to a standard deviation of 0.1 was set to prevent poor matching.

## Results

### Patients’ characteristics

**Fig. 1:**
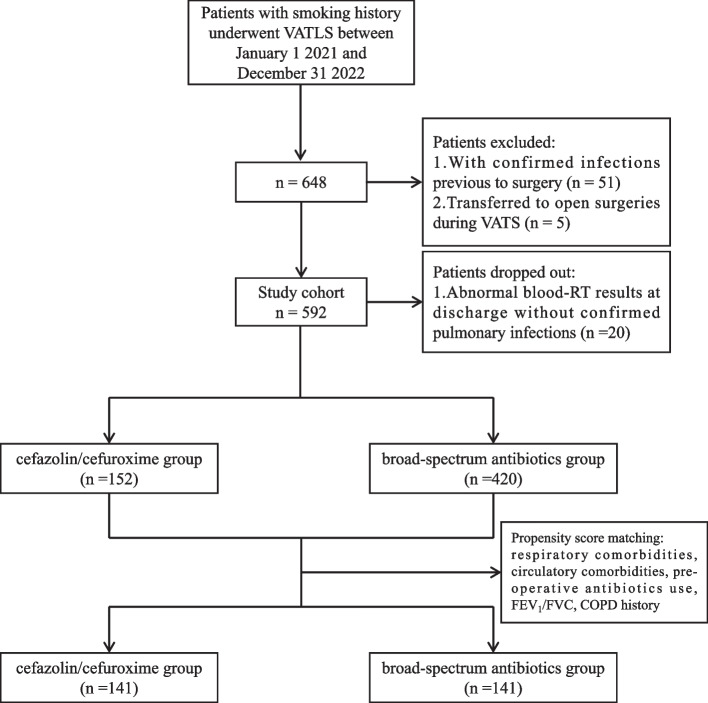
Flowchart of participants enrolled in the study. VATLS, video-assisted thoracoscopic lung surgery

In this analysis, 572 patients were included, of whom 152 received cefazolin/cefuroxime as the prophylactic antibiotic during peri-operative periods, while the other 420 received antibiotics other than the two. The flow chart was shown in Fig. 1. There were significant differences in respiratory comorbidities, circulatory comorbidities, pre-operative antibiotics use, the ratio of forced expiratory volume in 1 second (FEV_1_) to forced vital capacity (FVC) and COPD history between the two groups. After PSM with 141 in each group, the baseline characteristics were balanced. Details are shown in Table 1.

**Table 1:** Baseline characteristics of patients enrolled before and after propensity score matching

Variable	Before PSM			After PSM		
	With cefazolin/cefuroxime (*n* = 152)	With Other antibiotics (*n* = 420)	p	With cefazolin/cefuroxime (*n* = 141)	With Other antibiotics (*n* = 141)	p
Sex						
Female	0 (0.0)	8 (1.9)	0.190	0 (0.0)	5 (3.5)	0.071
Male	152 (100.0)	412 (98.1)		141 (100.0)	136 (96.5)	
Age, years (IQR)	61 (58, 66)	62 (55, 67)	0.995	61 (58, 66)	62 (57, 67)	0.521
BMI, kg/m^2^ (Mean)	23.78 ± 2.72	23.31 ± 2.95	0.088	23.68 ± 2.64	23.51 ± 3.14	0.638
Comorbidities						
Respiratory system						
Yes	19 (12.5)	82 (19.5)	**0.048**	19 (13.5)	16 (11.3)	0.588
No	133 (87.5)	338 (80.5)		122 (86.5)	125 (88.7)	
Circulatory system						
Yes	105 (69.1)	253 (60.2)	**0.050**	94 (66.7)	97 (68.8)	0.702
No	47 (30.9)	167 (39.8)		47 (33.3)	44 (31.2)	
Digestive system						
Yes	19 (12.5)	35 (83.)	0.132	19 (13.5)	10 (7.1)	0.108
No	133 (87.5)	385 (91.7)		122 (86.5)	131 (92.9)	
Urinary system						
Yes	16 (10.5)	30 (7.1)	0.189	16 (11.3)	11 (7.8)	0.312
No	136 (89.5)	390 (92.9)		125 (88.7)	130 (92.2)	
Endocrine system						
Yes	31 (20.4)	71 (16.9)	0.335	25 (17.7)	21 (14.9)	0.519
No	121 (79.6)	349 (83.1)		116 (82.3)	120 (85.1)	
Contagious disease						
Yes	4 (2.6)	7 (1.7)	0.691	4 (2.8)	3 (2.1)	1.000
No	148 (97.4)	413 (98.3)		137 (97.2)	138 (97.9)	
Number of cigarettes smoked, days (IQR)	20 (20, 20)	20 (10, 20)	0.126	20 (20, 20)	20 (10, 20)	0.068
Smoking time, years (IQR)	30 (20, 35)	30 (20, 40)	0.267	30 (20, 35)	30 (20, 40)	0.834
Quit smoking, years						
<1 year	104 (68.4)	320 (76.2)	0.061	99 (70.2)	103 (73.0)	0.597
≥1 years	48 (31.6)	100 (23.8)		42 (29.8)	38 (27.0)	
Pre-operative stay, days (IQR)	7.5 (5.0, 9.0)	7.0 (6.0, 10.0)	0.366	8 (5.5, 9.5)	7 (6, 9)	0.947
Pre-operative antibiotics use						
Yes	72 (47.4)	264 (62.9)	**0.001**	72 (51.1)	77 (54.6)	0.551
No	80 (52.6)	156 (37.1)		69 (49.9)	64 (45.4)	
FEV_1_/FVC, %						
≥70%	108 (71.1)	240 (57.1)	**0.003**	97 (68.8)	96 (68.1)	0.898
<70%	44 (28.9)	180 (42.9)		44 (31.2)	45 (31.9)	
COPD history						
Yes	6 (3.9)	38 (9.0)	**0.043**	6 (4.3)	6 (4.3)	1.000
No	146 (96.1)	382 (91.0)		135 (95.7)	135 (95.7)	
Baseline WBC count, × 10^9^/L (IQR)	6.00 (4.88, 6.80)	6.13 (4.83, 6.86)	0.680	6.02 (4.88, 6.80)	6.13 (4.83, 7.11)	0.899
Baseline neutrophils, % (IQR)	55.7 (52.0, 64.6)	58.1 (52.7, 62.5)	0.571	55.7 (51.8, 64.6)	58.1 (51.3, 63.2)	0.707
Baseline blood glucose, mmol/L (IQR)	4.88 (4.63, 5.44)	5.00 (4.68, 5.53)	0.165	4.87 (4.63, 5.41)	5.00 (4.60, 5.63)	0.172
Type of resection						
Wedge resection	27 (17.8)	49 (11.7)	0.137	25 (17.7)	22 (15.6)	0.558
Segmental resection	62 (40.8)	172 (41.0)		58 (41.1)	52 (36.9)	
Lobectomy	63 (41.4)	199 (47.4)		58 (41.1)	67 (47.5)	
Surgical site						
Left lung	69 (45.4)	173 (41.2)	0.369	63 (44.7)	63 (44.7)	1.000
Right lung	83 (54.6)	247 (58.8)		78 (55.3)	78 (55.3)	
Duration of VATLS, hours (IQR)	2.3 (1.9, 2.9)	2.4 (1.8, 3.0)	0.536	2.42 (1.95, 2.95)	2.43 (1.97, 3.26)	0.248

### Effects of cefazolin/cefuroxime for peri-operative prophylaxis in smoking patients

In terms of the primary outcome, the incidence of pulmonary infections confirmed by imaging examinations was 23.7% in the cefazolin/cefuroxime group, compared with 30.5% in the control group, but no significance was found between the two groups (*p* = 0.113).

As for secondary outcomes, the ratio of post-operative fever, the level of WBC count and ratio of neutrophils on the 3rd day were all without significance between the two groups. The incidence of post-operative fever was 10.5% in the cefazolin/cefuroxime group and 7.6% in the control group. The mean WBC count on the 3rd day was (9.07 ± 2.12) × 10^9^/L in the cefazolin/cefuroxime group, while (8.34 ± 2.39) × 10^9^/L in the control group. The mean ratio of blood neutrophils of patients received cefazolin/cefuroxime for prophylaxis was 73.49% ± 6.97% compared with 72.40% ± 8.54% of the control group.

For 408 patients without a confirmed pulmonary infection upon discharge, the times for post-operative blood-RT recovery were analyzed. The median times in cefazolin/cefuroxime group was 3 days, and 4 days in the control group, with inter-quartile ranges of (3,6) and (4,6), respectively. Similarly, no significant difference was found, either. After PSM, the results remained consistent as shown in Table 2.

**Table 2:** The outcomes of peri-operative prophylaxis with cefazolin/cefuroxime or other antibiotics during VATLS

Outcomes	Before PSM				After PSM			
	With cefazolin/cefuroxime (*n* = 152)	With Other antibiotics (*n* = 420)	Relative Risk (95% CI)	*p*	With cefazolin/cefuroxime (*n* = 141)	With Other antibiotics (*n* = 141)	Relative Risk (95% CI)	*p*
**Primary outcome**								
Pulmonary infection	36 (23.7)	128 (30.5)	0.777 (0.564 ~ 1.070)	0.113	36 (25.5)	43 (30.5)	0.837 (0.575 ~ 1.220)	0.353
**Secondary outcomes**								
Post-operative fever	16 (10.5)	32 (7.6)	1.382 (0.781 ~ 2.445)	0.268	16 (11.3)	13 (9.2)	1.231 (0.615 ~ 2.463)	0.556
WBC count on the 3rd day after surgery, × 10^9^/L	9.07 ± 2.12	8.34 ± 2.39		0.100	9.14 ± 2.15	8.75 ± 2.74		0.190
Neutrophils on the 3rd day after surgery, %	73.49 ± 6.97	72.40 ± 8.54		0.481	73.36 ± 7.09	73.09 ± 8.46		0.769
Time for post-operative blood-RT recovery^a^	3 (3,6)	4 (4, 6)		0.707	4 (3, 6)	4 (4, 6)		0.574

^a^Total number of patients was 408, with 116 patients in the cefazolin/cefuroxime group, and 292 in the control group

### Risk factors of pulmonary infections after VATLS in smoking patients

The unbalanced baseline variables were analyzed by univariate regression models and then included into the multivariate regression model. In the univariate analysis, all factors were found with no significant differences in association with post-operative pulmonary infections. In the multivariate analysis, when effects of respiratory comorbidities, circulatory comorbidities, pre-operative antibiotics, FEV_1_/FVC<70%, and COPD history were excluded, the association between post-operative pulmonary infections and prophylaxis with cefazolin/cefuroxime was still not significant (OR = 0.685, 95%CI 0.441 ~ 1.065, *p* = 0.093). Details were shown in Table 3.

**Table 3:** Univariate and multivariate logistic regression models for risk factors of pulmonary infections

Variable	Univariate regression model			Multivariate regression model		
	OR	95% CI	*P*	OR	95% CI	*P*
Prophylaxis with cefazolin/cefuroxime	0.708	0.462 ~ 1.086	0.114	0.685	0.441 ~ 1.065	0.093
Respiratory comorbidities	1.125	0.705 ~ 1.797	0.621	1.088	0.609 ~ 1.945	0.775
Circulatory comorbidities	1.314	0.897 ~ 1.925	0.160	1.378	0.927 ~ 2.048	0.113
Pre-operative antibiotics use	0.988	0.684 ~ 1.428	0.950	0.894	0.610 ~ 1.311	0.567
FEV_1_/FVC <70%	1.144	0.791 ~ 1.655	0.475	1.050	0.709 ~ 1.554	0.809
COPD history	1.176	0.607 ~ 2.280	0.631	1.041	0.445 ~ 2.438	0.926

## Discussion

This study conducted a retrospective cohort analysis to compare the effect of cefazolin/cefuroxime with broad-spectrum antibiotics in preventing post-operative pulmonary infections and other clinical outcomes in smoking patients undergoing VATLS. The incidence of post-operative pulmonary infections as the primary outcome between the cefazolin/cefuroxime prophylaxis group and the control group was found to be comparable with no statistical significance (23.7% vs 30.5%, RR = 0.777, 95%CI 0.564 ~ 1.070, *p* = 0.113). The ratio of post-operative fever between the two groups did not yield significant results as a secondary outcome, either (10.5% vs 7.6%, RR = 1.382, 95%CI 0.781 ~ 2.445, *P* = 0.268). Similarly, other secondary outcomes including WBC count, and ratio of neutrophils on the 3rd day post-surgery did not exhibit any significant differences. In determining the appropriate time for antibiotic cessation following surgical procedures, the recovery of blood-RT is often taken into consideration. In our study, the median time of post-operative blood-RT recovery in 408 patients without confirmed pulmonary infections was found to be similar between the two groups. To exclude the effects of the unbalanced baseline characteristics, the PSM method was also conducted and the results of patients’ outcomes remained consistent after PSM. Finally, both in univariate and multivariate regression models, the association between post-operative pulmonary infections and prophylaxis with cefazolin/cefuroxime were with no significance. These results collectively showed that peri-operative prophylaxis with cefazolin/cefuroxime in VATLS did not lead to more adverse outcomes especially post-operative pulmonary infections in smoking patients when compared with other broad-spectrum antibiotic prophylaxis regimens.

Whether post-operative pulmonary infections should be included into SSI is controversial. SSI is one of the most important complications after surgical procedures. The widely accepted definition of SSI described by the Surgical Wound Infection Task Force is “infection of the incision or organs/spaces manipulated during an operative intervention, including superficial incisional infection, deep incisional infection, and organ/space infection” [19]. Notably, remote post-operative infections such as pneumonia is often excluded. Guideline of antimicrobial prophylaxis in surgery published in 2013 described post-operative pneumonia alone and distinguished it from SSI even in thoracic surgeries [8]. But in another review, Stephanie et al. emphasized “SSI does not encompass remote postoperative infections such as pneumonia after non-thoracic surgery or urinary tract infections after non-urologic procedures” [20], which pointed out the particularity of post-operative pneumonia in thoracic surgery. After that, in 2017, the CDC updated the guideline of preventing SSI, however post-operative pulmonary infections were not discussed in this latest guideline [7]. As post-operative pulmonary infection has its unique characteristics in thoracic surgery and causes 20 to 30% death of lung surgery [10], antibiotic prophylaxis for this kind of infections is apparently with great importance.

Although minimally invasive surgeries, for example VATS, has decreased the risk of post-operative pulmonary infections [21], smoking is still a risk factor of increasing the possibility of infections. And this could explain why the infection rate of patients receiving VATLS in our study was much higher than the rate reported in the literature (23.7% and 30.5% vs 3%–7%), which included non-smokers [2–6]. The mechanism by which smoking leads to an increased susceptibility to bacterial infection involves multiple components. First, smoking alters the transcription program of basal cells, causing aberrant repair processes thus leading to airway remodelling [22]. Second, smoking causes the process associated with mucous hyper-secretion including mucous gland hypertrophy goblet cell hypertrophy and hyperplasia and this pathological change will lead to mucous plugging [23]. Third, smoking related inflammation of small airways induces immune cells infiltration [24]. These mechanisms result in airway bacterial colonization and airway injury, collectively leading to susceptibility to bacterial infections [24].

As describe above, prevention of post-operative pulmonary infection in thoracic surgery is still an important topic despite the ambiguity whether this infection should be included in SSI of thoracic surgery. Unfortunately, current guidelines do not have special recommendations on antibiotic prophylaxis of post-operative pulmonary infections for smoking populations. Clinical practice are usually based on local guidelines for preventing deep or superficial SSI. In this situation, first- and second-generation cephalosporins such as cefazolin and cefuroxime are often the choices in lung surgery including VATLS with the purpose of SSI preventing [10]. Though the efficacy of cefazolin and cefuroxime prophylaxis on preventing SSI has been studied in minimally invasive surgeries including laparoscopic [25–27] and thoracoscopic surgery [28], the effect for preventing post-operative pulmonary infections in thoracic surgery especially of smoking patients is unclear. Due to the high risk of pulmonary infections after VATLS in smoking patients and the lack of clear recommendations for antibiotic prophylaxis, this study filled the gap in this issue and verified that the efficacy of cefazolin/cefuroxime was comparable to other broad-spectrum antibiotic prophylaxis regimens. We also found that prophylaxis with cefazolin/cefuroxime would not increase post-operative fever, WBC count and ratio of neutrophils on the 3rd day after surgery. For those patients without confirmed pulmonary infections, the time for blood-RT recovery was not longer in the cefazolin/cefuroxime group. These results should increase doctors’ confidence in using prophylactic cefazolin/cefuroxime for smoking patients, as no significant differences were found in the major clinical outcomes between the two drugs and other broad-spectrum antibiotics.

The reason of the comparable incidence of pulmonary infections in smoking population receiving different prophylactic antibiotics could be explained as follows. First, despite changes in airway bacterial colonization, most reported isolated microorganisms from oral and nasopharyngeal in smoking populations were *Haemophilus influenzae*, *Streptococcus pneumoniae*, *Streptococcus viridans*, *Staphylococcus aureus* and *Klebsiella pneumoniae* [11, 16], which could be covered by cefazolin/cefuroxime if sensitive. For smoking populations and those with concurrent COPD, our concern regarding the colonization of *Pseudomonas aeruginosa* which could not be covered by cefazolin/cefuroxime is not fully supported by evidence. Therefore, in terms of antimicrobial spectrum, cefazolin/cefuroxime are sufficient. Second, minimally invasive surgical procedures, such VATS, causes less systemic immunological upset than open surgeries which may confer advantage in terms of maintaining immune defence mechanisms against bacterial attacks [29], and antibiotic prophylaxis only plays a secondary role in this defense mechanism.

Objectively, there are limitations in this study. First, we only tracked the outcomes of the study population during their hospital stays, and whether they developed pulmonary infections relevant to the VATLS after discharge was not known. According to Stephanie et al., SSI of organs included infections within 30 days [20], which was longer than patients’ total length of hospital stay in our study. Second, we lack the bacterial culture results of post-operative pulmonary infections, which makes it difficult for us to verify the causal relationship between the chosen prophylactic antibiotics and patients’ outcomes from a microbiological and pharmacological perspective. Third, it has been confirmed that important practices such as oral and pharyngeal disinfection [30], proper intraoperative anaesthesia techniques [31], as well as pre-operative and post-operative physical therapy [32], are effective measures for preventing post-operative pulmonary complications including infections. However, we could not obtain these information from the hospital information system and we could only assume that all patients undergoing VATLS were comparable in terms of these procedures. Future research should aim to collect more comprehensive information during the peri-operative periods for patients, and should include longer post-operative follow-up to validate the findings of this study.

## Conclusions

Based on this retrospective cohort study, we have not found an association between the use of cefazolin/cefuroxime, instead of broad-spectrum antibiotics, and the occurrence of adverse clinical outcomes including post-operative pulmonary infections, fever, and abnormal blood routine tests in smoking populations undergoing VATLS. Physicians should choose these narrow-spectrum antibiotics for peri-operative prophylaxis for smoking patients receiving this surgical procedure.

## Data Availability

All data generated or analyzed during this study are included in this published article.

## Abbreviations

VATS: Video-assisted thoracoscopic surgery
VATLS: Video-assisted thoracoscopic lung surgery
SSI: Surgical site infection
WBC: White blood cell
Blood-RT: Blood routine test
IQR: Inter-quartile range
PSM: Propensity score matching
COPD: Chronic obstructive pulmonary disease
FEV_1_: Forced expiratory volume in 1 second
FVC: Forced vital capacity
